# Interaction between W41 of the hepatitis B virus preS1 surface peptide and Y146/F274 of the cellular receptor molecule Na^+^/taurocholate co-transporting polypeptide is essential for virus entry

**DOI:** 10.1016/j.molpha.2025.100069

**Published:** 2025-08-08

**Authors:** Sebastian Kunz, Lena Soppa, Regina Leidolf, Anita Neubauer, Thomas Lütteke, Dieter Glebe, Joachim Geyer

**Affiliations:** 1 Institute of Pharmacology and Toxicology, Faculty of Veterinary Medicine, Biomedical Research Center Seltersberg, Justus Liebig University of Giessen, Giessen, Germany; 2 Institute of Medical Virology, National Reference Center for Hepatitis B Viruses and Hepatitis D Viruses, German Center for Infection Research (DZIF, Partner Site Giessen-Marburg-Langen), Faculty of Medicine, Justus Liebig University Giessen, Giessen, Germany; 3 Institute of Veterinary Physiology and Biochemistry, Faculty of Veterinary Medicine, Justus Liebig University of Giessen, Giessen, Germany

**Keywords:** Hepatitis B virus, Hepatitis D virus, preS1 peptide, Virus entry inhibitor, Na^+^/taurocholate co-transporting polypeptide, Structure-based drug development

## Abstract

The myristoylated preS1 domain (myr-preS1) of the hepatitis B virus (HBV) large surface protein is essential for binding to the receptor protein, Na^+^/taurocholate co-transporting polypeptide (NTCP), and for the subsequent internalization of the virus-receptor complex. NTCP, which is expressed in hepatocytes, plays a physiological role in hepatic bile acid transport. Recent cryo-electron microscopy structures of the myr-preS1-NTCP complex were used to analyze virus-receptor interactions at the molecular level. Several interaction domains with high binding energies have been identified, including the interaction between myr-preS1 tryptophan 41 (W41) and the NTCP residues tyrosine 146 and phenylalanine 274 (Y146/F274), which are located at a considerable distance from the physiological bile acid binding sites of NTCP. The Y146A/F274A mutant of NTCP retained bile acid transport function but showed a reduced myr-preS1 binding. The W41G myr-preS1 mutant exhibited reduced binding to wild-type NTCP. The Y146A/F274A NTCP mutant did not support in vitro HBV infection, and the W41G myr-preS1 peptide was less effective in blocking infection compared with the wild-type myr-preS1 peptide. In conclusion, the myr-preS1-W41/NTCP-Y146/F274 interaction site, characterized by high binding energy, is essential for HBV entry into hepatocytes. Because this domain is spatially distinct from the bile acid binding and translocation sites of NTCP, it presents an attractive receptor target site for structure-based development of virus-selective HBV entry inhibitors that preserve the physiological bile acid transport function of NTCP.

**Significance Statement:**

This study identified and validated a high energy interaction site between the hepatitis B virus and its receptor Na^+^/taurocholate co-transporting polypeptide that can be used for structure-based drug design of virus entry inhibitors.

## 1 Introduction

The hepatitis B virus (HBV) belongs to the family of *Hepadnaviridae*. Its DNA genome encodes 3 surface proteins (HBs), namely small HBs, middle HBs, and large HBs.^1^ The hepatitis D virus (HDV) is a small negative-sense RNA satellite virus and depends on the HBV envelope for entry into hepatocytes, assembly, and secretion of newly formed virions.^2^ In 2012, the Na^+^/taurocholate co-transporting polypeptide (NTCP, gene symbol *SLC10A1*) has been identified as the high-affinity hepatic entry receptor for HBV and HDV.^3^ HBV/HDV attachment to NTCP occurs via the myristoylated preS1 domain (myr-preS1) of the large HBs^4^^,^^5^ and triggers the internalization of the virus-receptor complex in an endocytosis-dependent manner.^6^ NTCP is specifically expressed at the basolateral membrane of hepatocytes and is essential for the hepatic uptake of bile acids during their enterohepatic circulation.^7^^,^^8^ Based on these findings, pharmacological inhibition of HBV/HDV binding to NTCP is an attractive strategy for the development of antiviral drugs acting as HBV/HDV entry inhibitors.^9^, ^10^, ^11^ The myr-preS1 peptide analog bulevirtide (Hepcludex, formerly known as myrcludex B) has been approved in July 2023 as the first-in-class HBV/HDV entry inhibitor for the treatment of chronic HDV infection in Europe and currently is being tested in clinical trials on patients with HBV infection or HBV/HDV coinfection.^2^^,^^12^, ^13^, ^14^ However, bulevirtide as a peptide-based drug must be administered by daily subcutaneous injections. Bulevirtide is generally well tolerated, but because of the nonselective mode of NTCP inhibition is associated with an increase of plasma bile acid levels with unclear long-term safety.^15^^,^^16^ Over the past decade, there have been intensive efforts to identify orally bioavailable NTCP inhibitors. Several small molecules have already demonstrated the ability to inhibit NTCP and prevent HBV/HDV infection of NTCP-expressing hepatoma cells. However, most of these compounds block NTCP in a nonselective manner, impairing not only virus binding to NTCP but also its physiological bile acid transport function. These molecules include bile acid derivatives such as DBA-41 or obeticholic acid,^17^^,^^18^ steroid-based compounds such as ZINC000253533654,^19^ plant-derived compounds such as vanitaracin A, exophillic acid, or betulinic acid,^20^, ^21^, ^22^ as well as approved drugs such as irbesartan, ritonavir, cyclosporine A, ezetimibe, or rosiglitazone.^23^, ^24^, ^25^, ^26^ Only a few studies have identified compounds with a predominantly antiviral effect over the bile acid transport inhibition, namely certain derivatives of betulinic acid^21^ and cyclosporine A.^27^ The first oral NTCP inhibitors have already been tested in preclinical and clinical studies as novel HBV/HDV entry inhibitors, namely A2342^28^ and FRI-231.^29^

Only 2 years ago, 4 independent studies reported cryo-electron microscopy (cryo-EM) structures of human NTCP.^30^, ^31^, ^32^, ^33^ They consistently revealed 9 transmembrane domains (TMs) that are arranged in a panel domain (formed by TM 1, 5, and 6) and a core domain (formed by TM 2–4 and 7–9) and showed a crossing motif (X-motif) formed by TM 3 and 8 near to the sodium binding sites. In the structure with the highest resolution, 2 distinct binding sites for bile acid molecules have been identified that are located within a tunnel-like structure at the interface between the core and the panel domains.^32^ Moreover, very recently, 2 independent studies resolved structures for the NTCP receptor in complex with the myr-preS1 peptide^34^ or the myr-preS1 analog bulevirtide.^35^ Both structures revealed interaction sites of myr-preS1 with the tunnel and surface structures of NTCP. These studies now enable the structure-based development of NTCP inhibitors that may act as HBV/HDV entry inhibitors.

In the present exploratory study, we aimed to identify structural domains of NTCP that would be appropriate for structure-based development of HBV/HDV entry inhibitors that selectively block virus binding to NTCP without tackling the physiological bile acid transport function of NTCP. Therefore, we focused on the surface region of NTCP where the preS1-NTCP interactions are most distant from the bile acid substrate binding sites.^32^^,^^35^ Interestingly, amino acid Y146 of NTCP, among others, fulfills this condition and has already been identified as essential for the HBV receptor function of NTCP in a previous study.^36^ Based on recent structural information, we identified a strong virus-receptor interaction site at the molecular level, involving noncovalent *π*–*π* stacking interactions between Y146 and F274 of NTCP, bridging W41 of the myr-preS1 peptide. Y146A/F274A double mutation of NTCP completely abolished in vitro HBV infection of transfected human hepatoma cells (HepG2). This data makes the myr-preS1-W41/NTCP-Y146/F274 interaction site an attractive target site for virus-selective HBV/HDV entry inhibitors.

## 2 Materials and methods

### 2.1 Chemicals

All the chemicals, unless otherwise stated, were purchased from Sigma-Aldrich. Radio-labeled [^3^H]taurocholic acid ([^3^H]TCA, 10 Ci/mmol) was purchased from PerkinElmer Life Sciences. The W41G mutant myr-preS1 peptide was obtained from ProteoGenix S.A.S. The fluorescent 4-nitrobenzo-2-oxa-1,3-diazole (NBD)-coupled bile acid derivatives 3*β*-NBD-TCA^37^ and 3*α*-NBD-TCA^38^ were used as previously reported.

### 2.2 Molecular dynamics simulation of the bulevirtide-NTCP interaction

All in silico predictions were done by YASARA Structure (YASARA Biosciences GmbH). For visualization of the cryo-EM structure of human NTCP-bulevirtide complex^35^ (Protein Data Bank [PDB] 8RQF), the standard settings of the YASARA viewer were used. For integrating the object into a membrane and in a water filled simulation cell, the standard defined macroscript (md_runmembrane.mcr) was used in the YASARA command. This macro sets up and runs a simulation of a membrane protein. It scans the protein for secondary structure elements with hydrophobic surface residues, orients them accordingly and embeds them in a membrane of adjustable lipid composition. Then, a 250 ps equilibration simulation was run, which ensured that the membrane adapts to the newly embedded protein. Subsequently, 20 independent molecular dynamic (MD) simulations were run, each over 100 ps. The number of contacts between myr-preS1 and NTCP have been collected and summed for each run. The simulation was run at physiological conditions of 0.15 M NaCl (physiological condition), pressure of 1 bar, pH of 7.4, and a constant temperature of 309.65 K (36.5 °C). The lipid composition of the used model membrane was 100% phosphatidylethanolamine without cholesterol as a simple bilayer. The chosen conditions of the simulation were AMBER14 force field (Assisted Model Building and Energy Refinement) and periodic boundaries for the protein. In addition, the following force field settings of YASARA were used: LIPID14 for the phospholipids, TIP3P for the water model and Li and Merz for the ion force field parameters. For analyzing the molecular interactions between 2 residues the standard defined macroscript (md_analyze.mcr) of YASARA command was used. This macro analyzes a simulation and creates a detailed report with many plots, for example, energies, root mean square deviations (RMSDs), and hydrogen bonds (HB). It also identifies the main ligand and provides ligand-specific data. Mutations in the preS1 and NTCP sequences were introduced via the swap (edit) command.

### 2.3 Cell culture

T-Rex Flp-In human embryonic kidney (HEK293) cells (Thermo Fisher Scientific; RRID CVCL_0045) were grown in 1:1 Dulbecco’s modified Eagle medium (DMEM)/Ham’s F12 (Gibco). Both media were supplemented with 10% fetal calf serum (Gibco), L-glutamine 4 mM (Gibco), penicillin 100 U/mL (Carl Roth), and streptomycin 100 *μ*g/mL (Carl Roth). Stably transfected T-Rex Flp-In HEK293 cells were selected with 100 *μ*g/mL hygromycin (Carl Roth). Expression of the recombinant NTCP proteins was induced with 5 *μ*g/mL tetracycline treatment for 72 hours. All cell lines were cultivated in 5% CO_2_ atmosphere at 37 °C. Cell lines were routinely checked for possible mycoplasma contamination.

### 2.4 Side-directed mutagenesis and cell transfection

Site-directed mutagenesis was performed on the cloned human NTCP-FLAG construct in the pcDNA5/FRT/TO vector^36^ using oligonucleotide primers synthesized from Metabion International AG. The following oligonucleotide primers were used: Y146A mutation, 5′-CAT CTA CTC CAG GGG GAT CGC TGA TGG GGA CCT GAA GGA C-3′ forward and 5′-GTC CTT CAG GTC CCC ATC AGC GAT CCC CCT GGA GTA GAT G-3′ reverse; F274A mutation, 5′-ATC CTC AAT GTG GCC GCT CCA CCT GAA GTC AT-3′ forward and 5′-ATG ACT TCA GGT GGA GCG GCC ACA TTG AGG AT-3′ reverse. The generated mutants were sequence-verified by Sanger sequencing (Microsynth AG). The constructs were used for the generation of a NTCP Y146A/F274A double mutant tetracycline-inducible stable cell line based on T-Rex Flp-In HEK293 cells as reported.^36^ Cell lines are referred to as wild-type NTCP (NTCPwt)-HEK293 for the NTCPwt and NTCP-Y146A/F274A-HEK293 for the double mutant. All transfections were performed using Lipofectamine 2000 according to manufacturer’s instructions (Invitrogen).

### 2.5 Immunofluorescence microscopy

NTCPwt-HEK293 and NTCP-Y146A/F274A-HEK293 cells were grown on poly-L-lysine coated 8-well *μ*-slides (IBIDI). Cells were treated with 5 *μ*g/mL tetracycline (Carl Roth) for 72 hours, fixed with 2% paraformaldehyde and blocked with blocking buffer, containing 5% bovine serum albumin in phosphate-buffered saline (PBS, containing 137 mM NaCl, 2.7 mM KCl, 1.5 mM KH_2_PO_4_, and 7.3 mM Na_2_HPO_4_, at pH 7.4), for 45 minutes at room temperature (RT). Then, cells were incubated at 4 °C overnight with a rabbit anti-FLAG antibody (1:2000 dilution, Sigma-Aldrich, Cat. #F7425) in blocking buffer, followed by labeling with AlexaFluor488-conjugated anti-rabbit secondary antibody (1:200 Invitrogen, RRID AB_143165). Nuclei were stained with Hoechst 33342. Z-stack cell imaging was performed at RT on an inverted Leica DMI6000 B fluorescent microscope and analysis of the fluorescence images was performed with the LAS X software (Leica).

### 2.6 Native membrane protein extraction and deglycosylation

Extraction of membrane proteins was performed with the ProteoExtract Native Membrane Protein Extraction Kit (Sigma-Aldrich) from confluent HEK293 monolayers with ∼10 × 10^6^ cells. During the extraction procedure, wash and extraction buffers I and II were kept on ice and protease inhibitor cocktail at RT. The growth medium was removed without disturbing the cell monolayer. Cells were carefully washed twice with 2 mL ice cold wash buffer. Then, 10 *μ*L of the protease inhibitor cocktail and 2 mL ice cold extraction buffer I were added to the cell plate. The extraction buffer I was incubated for 10 minutes at 4 °C. The supernatant was collected (cytoplasmatic fraction). Then, 5 *μ*L protease Inhibitor cocktail and 1 mL ice cold extraction buffer II were added to the cell plates. The extraction buffer II incubated for 30 minutes at 4 °C. Supernatants representing the membrane protein fraction were transferred to a separate collecting tube. For deglycosylation of the NTCP proteins of NTCPwt-HEK293 and NTCP-Y146A/F274A-HEK293 cells with PNGase F (New England Biolabs, P0704S), the cytoplasmatic and the membrane fractions were used. For each sample a total reaction volume of 70 *μ*L was used by adding 7 *μ*L GlycoBuffer 2 (10×), 7 *μ*L 10% NP-40, 5 *μ*L H_2_O, and 1 *μ*L PNGase F to 50 *μ*L of the protein fraction. The enzymatic digestion incubated at 37 °C for 1 hour.

### 2.7 Western blotting

Samples from native membrane protein extraction followed by PNGase deglycosylation were mixed with Laemmli sample buffer containing 2% SDS (Carl Roth), 10% glycerol (Carl Roth), 0.002% bromophenol blue (Merck), 62.5 mM Tris-HCl (Carl Roth), and 5% 2-mercaptoethanol (Carl Roth) and heated at 95 °C for 10 minutes. Then, samples were separated on a 10% SDS polyacrylamide gel under reducing conditions, followed by electro transfer to a Roti-PVDF membrane (Carl Roth). After blocking in TBS-T buffer (0.05% Tween-20 in PBS) (Carl Roth) containing 5% nonfat milk (Carl Roth), the membranes were probed with anti-FLAG rabbit polyclonal antibody (1:2000 dilution, Sigma-Aldrich, Cat. #F7425, RRID AB_439687) overnight at 4 °C. Staining against glyceraldehyde-3-phosphate dehydrogenase served as loading control and was performed using goat anti-glyceraldehyde-3-phosphate dehydrogenase antibody (1:1000 dilution, Sigma-Aldrich, Cat. #SAB2500450). Subsequently, the membranes were incubated with peroxidase-labeled anti-rabbit and anti-goat secondary antibodies (1:3000 dilution, Thermo Fisher Scientific, Cat. #31460 and Cat. #31431, respectively). Membranes were finally incubated with the ECL Plus Kit (Amersham Biosciences) and signals were acquired using the ChemiDoc Image System and Image Lab software (Bio-Rad Laboratories).

### 2.8 Bile acid transport assay

For quantitative transport experiments, cells were seeded onto poly-L-lysine coated 96-well plates and grown to confluence over 72 hours at 37 °C. Then, cells were washed 3 times with PBS followed by preincubation in sodium transport buffer containing 142.9 mM NaCl, 4.7 mM KCl, 1.2 mM MgSO_4_, 1.2 mM KH_2_PO_4_, 1.8 mM CaCl_2_, and 20 mM HEPES (pH 7.4). For transport assays under sodium-free conditions (-Na^+^), sodium chloride of the sodium transport buffer was substituted with equimolar concentrations of choline chloride. Transport assays were started by adding NBD-TCA or [^3^H]TCA to the transport buffer, followed by incubation at 37 °C. For inhibition experiments, cells were preincubated at 37 °C with 80 *μ*L containing the respective myr-preS1 inhibitor at increasing concentrations. Then, transport experiments were started by adding 20 *μ*L sodium transport buffer containing [^3^H]TCA. All transport experiments were stopped after 10 minutes by aspirating the transport buffer and washing the cells twice with ice-cold PBS. Cell-based fluorescence was directly analyzed by Glomax fluorescence reader (Promega) at 488 nm. For radioactive measurements the plates were kept cool until adding the lysis buffer (containing 1% sodium dodecyl sulfate and 1 N NaOH). Cell-associated radioactivity was quantified by liquid scintillation counting in a Packard Microplate Scintillation Counter TopCount NXT (Packard Instrument Company).

### 2.9 Myr-preS1 peptide binding assay

To study the HBV receptor function of NTCP, NTCPwt-HEK293 and NTCP-Y146A/F274A-HEK293 cells were incubated with the C-terminally Alexa 568 fluorophore-coupled myr-preS1 peptide (further referred to as myr-preS1-AX568), which consists of amino acids 2 to 48 of the large HBV genotype D3 surface protein (Biosynthesis). Briefly, cells were washed 3 times with DMEM and then incubated with 5 nM myr-preS1-AX568 peptide in DMEM for 10 minutes at 37 °C. After extensive washing, cells were fixed with 2% paraformaldehyde (Carl Roth), washed again, and blocked with 5% bovine serum albumin in PBS for 1 hour at RT. Nuclear staining was performed with Hoechst 33342 (1 mg/mL) for 10 minutes at RT. Myr-preS1-AX568 peptide binding was analyzed using Leica DMI6000 B inverted fluorescent microscope and LAS X software, or fluorescence was quantified with Spark multimode microplate reader (Spark Multimode Microplate, Tecan) (excitation: 570 nm, emission: 615 nm). Peptide binding experiments were also conducted with a similar myr-preS1 peptide of HBV subgenotype D3 that was, however, tritium labeled (1 mCi/mL, Pharmaron, further referred to as [^3^H]preS1), as reported before.^19^ The medium was replaced with 80 *μ*L of DMEM containing the respective myr-preS1 inhibitor peptide or solvent alone (100% uptake/binding control), and cells were further incubated for 5 minutes at 37 °C. Binding of [^3^H]preS1 was initiated by adding 20 *μ*L of DMEM containing 25 nM [^3^H]preS1 (final concentration: 5 nM). After 10 minutes, experiments were stopped by washing twice with ice-cold PBS. Cell-associated radioactivity was quantified by liquid scintillation counting in a Packard Microplate Scintillation Counter TopCount NXT.

### 2.10 In vitro HBV infection of NTCP-transfected HepG2 cells

HepG2 cells (Clontech; RRID CVCL_KT01) were transiently transfected with the NTCPwt and NTCP-Y146A/F274A constructs using X-tremeGENE HP Transfection Reagent (Roche Diagnostics GmbH) according to the manufacturer’s protocol. Cells were incubated in Hepatocyte Growth Medium, which contained 2% DMSO, 5 *μ*g/mL caspofungin, and 1 *μ*g/mL fungizone. The corresponding Hepatocyte Growth Medium was composed of Williams’ Medium E (Gibco/Thermo Fisher Scientific) supplemented with 2% bovine serum albumin (Carl Roth), 1x GlutaMAX Supplement (Gibco/Thermo Fisher Scientific), 100 *μ*g/mL gentamicin (Gibco/Thermo Fisher Scientific), 10 nM dexamethasone (Merck), 1 mM sodium pyruvate (Gibco/Thermo Fisher Scientific), and 1x Insulin-Transferrin-Selenium (Gibco/Thermo Fisher Scientific), as reported.^39^ Immunofluorescence staining of NTCP expression of the transfected HepG2 cells was done with mouse monoclonal anti-FLAG M2 primary antibody (Merck, RRID AB_262044) and a goat anti-mouse IgG (H+L) secondary antibody conjugated with Alexa Fluor 488 (Thermo Fisher Scientific, RRID AB_2534057), along with 4′,6-diamidino-2-phenylindole staining to determine the number of nuclei for further comparison of transfection efficiency. In addition, transport experiments were performed with NBD-TCA as reported.^39^ The percentage of NBD-TCA transporting and anti-NTCP-FLAG immunoreactive cells was analyzed using the ImageXpress Pico automated cell imaging system (Molecular Devices LLC). Before HBV infection, NTCP-expressing HepG2 cells were preincubated with 10, 100, and 500 nM of wild-type or mutant myr-preS1 peptides of HBV genotype D3 for 1 hour. Subsequently, cells were infected with cell-culture-produced HBV (genotype D3) derived from a stably HBV-genome transfected HepG2 cell line as outlined before,^40^ using the virus inoculum of 2 × 10^9^ HBV genomes/well of a 48-well plate. Following infection, the cell culture medium (Hepatocyte Growth Medium supplemented with 3 *μ*g/mL doxycycline, 2% DMSO, 5 *μ*g/mL caspofungin, and 1 *μ*g/mL fungizone) was renewed every 2 to 3 days. Using the supernatants collected on day 10 post infection, secreted HBeAg was determined using the diagnostic HBeAg Architect assay (Abbott).

### 2.11 Data analysis and statistics

All transport and inhibition graphs were generated with GraphPad Prism (GraphPad). Determination of IC_50_ was done by nonlinear regression analysis using the equation log(inhibitor) vs response – variable slope settings to fit the data. All data points of the IC_50_ curves represent means ± SD of quadruplicate determinations. Statistical analysis was performed as indicated in the figure legends with GraphPad Prism. The sample sizes for all experiments were prespecified before the study began as outlined in detail in the respective figure legends. As the present study is an exploratory study, *P* values must be interpreted as descriptive.

## 3 Results

### 3.1 Molecular interaction sites between myr-preS1 and NTCP based on the cryo-EM structure of the bulevirtide-NTCP complex

**Fig. 1:**
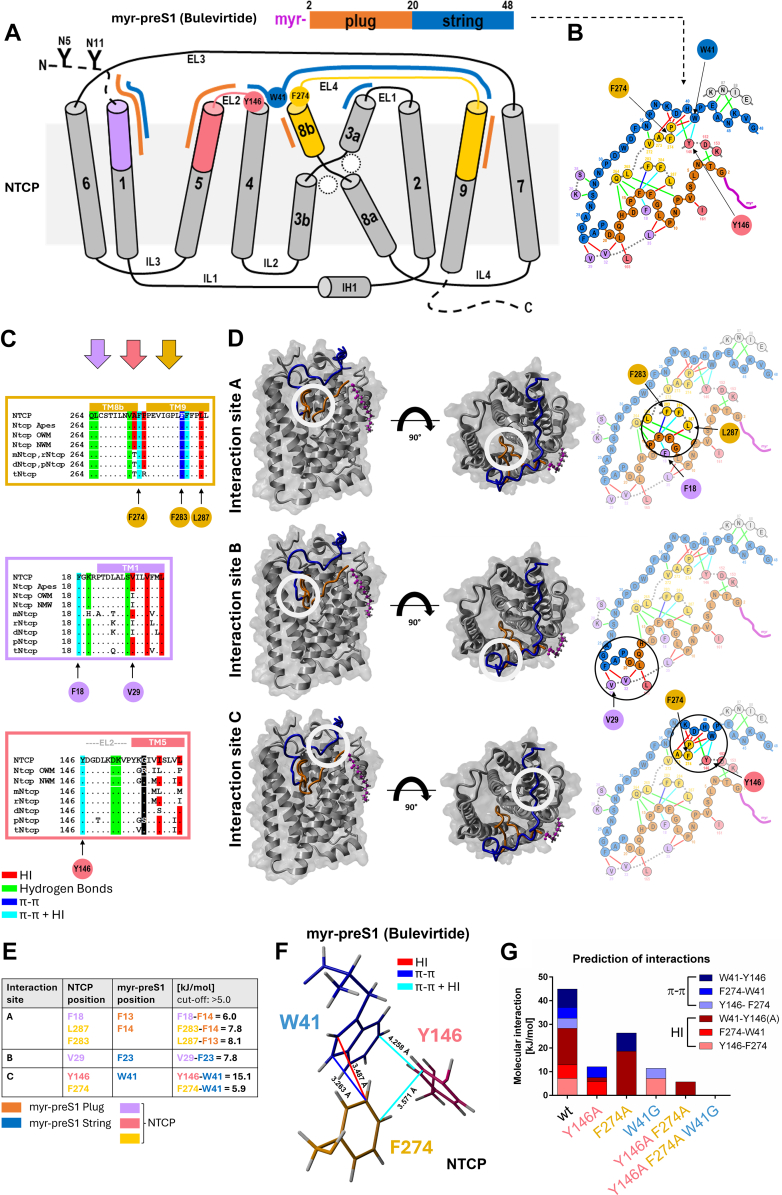
Molecular interactions between the HBV myr-preS1 peptide analog bulevirtide and the NTCP receptor. (A) Transmembrane domain model of NTCP showing the structural organization of NTCP in 9 TM, connected by extracellular loops (EL) and intracellular loops (IL). N-glycosylation sites at N5 and N11 as well as the position of intracellular helix 1 (IH1) are indicated. The diagram illustrates the interaction sites of NTCP with the myr-preS1 peptide analog bulevirtide based on the recent cryo-EM structure of the NTCP-bulevirtide complex^35^ (PDB 8RQF). The myr-preS1 structure is separated into a plug structure (orange) and a string structure (blue). The most relevant interaction sites at NTCP are colored in purple (TM1), red (TM5 and EL2) and yellow (TM8b, EL4, and TM9), respectively. (B) Molecular interactions between NTCP and bulevirtide at the amino acid level. The amino acids are colored corresponding to the domain topology in (A). Molecular interactions were categorized in HB (green lines), HIs (red lines), *π–π* interactions (dark blue lines), and *π–π* plus HIs (light blue lines). (C) Multispecies sequence alignment of the most relevant NTCP interaction sites covering TM8b, EL4, and TM9 (yellow), TM1 (purple), and TM5 and EL2 (red). Molecular interactions (HB, *π–π*, and HI) are indicated for the respective species NTCP/Ntcp sequences. (D) Localization of the most relevant interaction sites A–C in the 3D model of the NTCP-bulevirtide complex and molecular interactions at the amino acid level. (E) Interaction energy predictions for interaction sites A–C by YASARA based on the cryo-EM structure of the NTCP-bulevirtide complex. The following YASARA command was used: show interactions in distance below 5 Å, include contacts, hydrophobic bonds and other interactions such as *π–π*, cation–*π*, and ionic interactions. The command was defined to show any interactions between all amino acids of NTCP and all amino acids of bulevirtide. Interaction energies for interaction sites A–C are presented in kJ/mol for the most relevant amino acid interactions with cut off >5.0 kJ/mol. (F) Detailed structure of the proposed aromatic amino acid triangle structure between Y146 and F274 of NTCP and W41 of the myr-preS1 analog bulevirtide (interaction site C). (G) Interaction energy calculation for interaction site C for the indicated NTCP and myr-preS1 mutants. HIs are colored red and *π–π* interactions are shown in blue. Bars represent the sum of all binding energies for the indicated wild-type and mutant NTCP and bulevirtide proteins, respectively.

Based on the recent cryo-EM structure of the myr-preS1 peptide analog bulevirtide bound to human NTCP^35^ (PDB 8RQF), we reanalyzed the molecular interactions between both components using the MD tool YASARA. Bulevirtide represents a myr-preS1 peptide analog, and its sequence is based on the 2 to 48 amino acids of the HBV myr-preS1 peptide of genotype C.^4^ For our analyses, the bulevirtide-NTCP complex was integrated into an artificial membrane and binding energies between bulevirtide and NTCP were determined after 250 ps equilibration. As an output, hydrophobic interactions (HIs), hydrogen bonds (HB), and *π*–*π* interactions between individual amino acids were obtained. Figure 1A shows a 9-transmembrane model of human NTCP as reported before^35^ and indicates the sites of interaction between the plug and string structures of bulevirtide with respective structures of NTCP. The so-called plug structure of bulevirtide, representing amino acids 2 to 20 of myr-preS1, interacts with the extracellular parts of TM 1, 5, 8b, and 9 of NTCP. The so-called bulevirtide string structure, representing amino acids 21 to 48 of myr-preS1, interacts with the extracellular part of TM 1, 4, 8b, 3a, and 9, as well as with the extracellular loops (EL) 2 and 4 of NTCP. These molecular interactions are visualized in Fig. 1B at the amino acid level, whereby the coloring of the respective domains of bulevirtide and NTCP corresponds to the regions and domains shown in the overview of Fig. 1A. In this diagram, HI between individual amino acids are indicated by red lines, HB are illustrated in green, and *π*–*π* interactions by dark blue. Interactions including HI plus *π–π* interactions are indicated by light blue lines. Interestingly, as shown by multispecies alignment many of these interactions were at highly conserved amino acid positions between human NTCP and Ntcps from various animal species, including apes, old world monkeys (OWM), new world monkeys (NWM), mouse (mNtcp), rat (rNtcp), dog (dNtcp), pig (pNtcp), and tree shrew (*Tupaia belangeri*, tNtcp) (Fig. 1C).

Interactions at highly conserved positions were detected at Q264 (HB), L265 (HB), V272 (HB), F274 (*π–π* and HI), F283 (*π–π*), F284 (*π–π* and HI), L287 (HI), F18 (*π–π* and HI), S28 (HB), V32 (HI), L35 (HI), Y146 (*π–π* and HI), D152 (HB), and K253 (HB). Of note NTCP G158 in TM 5 is converted to R158 in all OWM and this amino acid substitution is responsible for their nonsusceptible phenotype for HBV infection.^39^ From all these interactions between NTCP and bulevirtide, 3 interaction hot spots with particularly high molecular interaction energies were identified and referred to as interaction sites A to C in Fig. 1D. Interaction site A includes strong interactions between F18 of the NTCP N-terminus as well as L287 and F283 of TM9 with F13 and F14 of the plug structure of bulevirtide (Fig. 1E). Interaction site B includes high energy interaction between NTCP V29 of TM1 and F23 of the string structure of bulevirtide. Finally, interaction site C is formed between Y146 of EL2 and F274 of TM8b of NTCP with W41 of the bulevirtide string. The total binding energy of interaction site C is at 45 kJ/mol and is built from a triangle of *π–π* and HI interactions between the aromatic rings of Y146, F274 and the side chain of W41, respectively (Fig. 1, F and G).

Subsequent MD simulations confirmed that all 3 components of this interaction triangle are essential for this strong interaction. For these calculations Y146A or F274A mutants of NTCP as well as a W41G mutant of bulevirtide were used for MD simulation and the calculated binding energies are depicted in Fig. 1G. Binding energies are separated for *π–π* interactions (colored in blue) and HI (colored in red). Mutation of each individual component of this binding triangle and even more pronounced Y146A/F274A double mutation of NTCP or triple mutation with additional W41G mutation of bulevirtide significantly reduced the overall binding energies. In detail, among the mutants, Y146A is predicted to lose 32.7 kJ/mol of binding energy. For F274A and W41G mutations the binding energy dropped by 22.7 and 37.1 kJ/mol, respectively.

### 3.2 Time-resolved analysis of the molecular interaction sites between myr-preS1 and NTCP by MD simulation

**Fig. 2:**
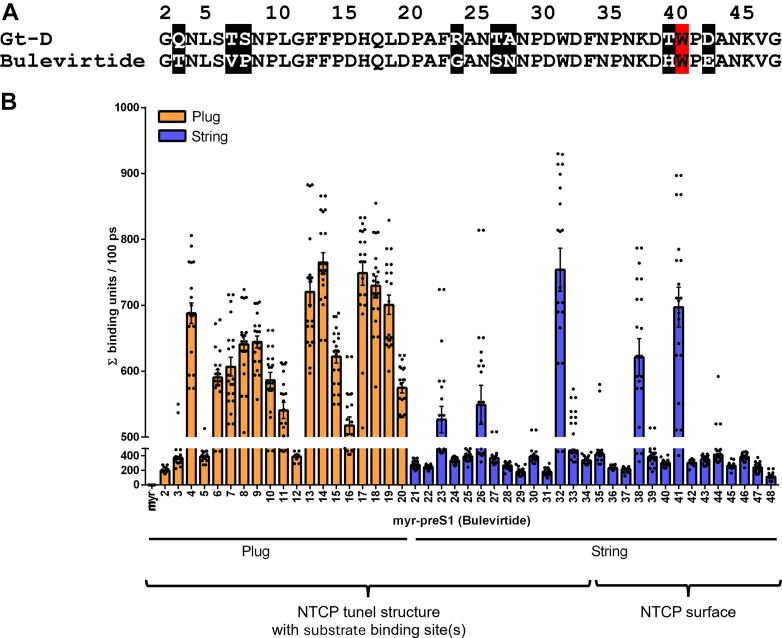
Molecular interactions between bulevirtide and NTCP in a series of 20 independent MD simulations. (A) Alignment of bulevirtide and the myr-preS1 peptide of genotype D covering the plug structure (amino acids 2–20) and string structure (amino acids 21–48). Sequence differences are shaded. (B) Based on the cryo-EM structure of the bulevirtide-NTCP complex^35^ (PDB 8RQF), 20 MD simulations (Macros: [md_runmembrane.mcr] and [md_analyze.mcr]) were performed with YASARA, each over 100 ps. For each MD simulation, all interactions (counted as 1–15 binding units per ps) between bulevirtide and NTCP were summed up for the whole simulation time and are presented at the *y*–axis. Bars represent mean ± SEM of 20 MD simulations.

Closer analysis of the time-resolved molecular interactions between NTCP and bulevirtide was done by 20 independent YASARA MD runs, each over 100 ps and all started from the membrane-embedded and equilibrated protein complex^35^ (PDB 8RQF). Figure 2A shows a sequence alignment of bulevirtide and myr-preS1 peptide of genotype D that was later used for in vitro infection experiments. Amino acids 2 to 20 represent the plug structure and amino acids 21 to 48 belong to the string structure of bulevirtide. Figure 2B depicts the number of contacts per simulation for all amino acids of bulevirtide with NTCP over the time of 100 ps. Based on the MD simulation, the following amino acids of bulevirtide dominantly interact with NTCP: N4, F13, F14, H17, Q18, and L19 from the plug structure, as well as W32 and W41 from the string structure. Among these amino acids, only W41 is considered not to interact with the bile acid substrate binding at the tunnel structure of NTCP. Therefore, the present study focused on the role of myr-preS1 W41 for the HBV virus interaction with NTCP. Based on this MD simulation and the strong binding energy of the NTCP Y146/F274–myr-preS1 W41 triangle structure we aimed to validate the functional relevance of this interaction site.

### 3.3 Experimental validation of the intact bile acid transport function and reduced myr-preS1 binding capability of the Y146A/F274A mutant NTCP

**Fig. 3:**
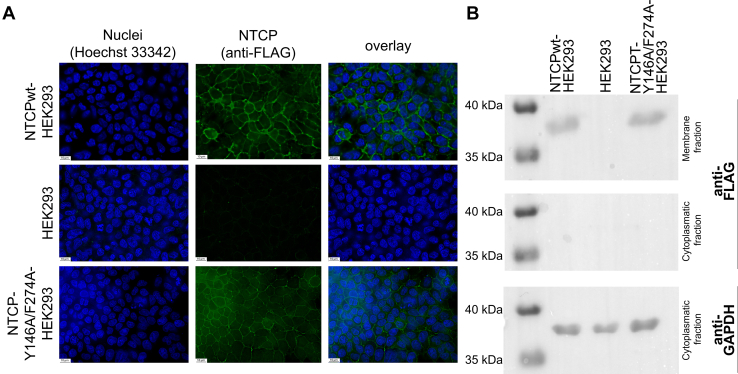
Plasma membrane expression of the NTCP-Y146A/F274A mutant. (A) Immunofluorescence staining with the anti-FLAG antibody (green fluorescence) localized the NTCP-Y146A/F274A mutant protein to the plasma membrane of HEK293 cells comparable to the localization of the NTCPwt protein. Nuclei were stained with Hoechst 33342 (blue). Images represent maximum projections of z-stacks at 63× magnification after deconvolution. Scale bar, 10 *μ*m. (B) Western blot expression analysis of the NTCPwt and NTCP-Y146A/F274A mutant proteins. Proteins were extracted from the respective NTCPwt-HEK293, NTCP-Y146A/F274A-HEK293, or nontransfected HEK293 cells with the ProteoExtract membrane protein extraction kit and were subjected to enzymatic deglycosylation with PNGase before polyacrylamide gel separation. Expression of GAPDH served as loading control. No NTCP signals were detected in the cytoplasmic fraction.

As a first step of experimental validation, we introduced mutations at positions Y146 and F274 of human NTCP (Y146A and F274A) and generated cell lines stably express the resulting double mutant NTCP protein. As shown in Fig. 3A, immunofluorescence microscopy clearly detected the NTCP Y146A/F274A mutant protein in the plasma membrane of HEK293 cells comparable to the NTCPwt protein. Western blot analysis of both cell lines with an anti-FLAG antibody was performed after membrane protein extraction and deglycosylation by PNGase F and revealed bands of comparable molecular weight and intensity for the mutant and NTCPwt proteins (Fig. 3B). In contrast, both NTCP proteins were not detected in the cytoplasmic fractions of the respective HEK293 cells lines.

**Fig. 4:**
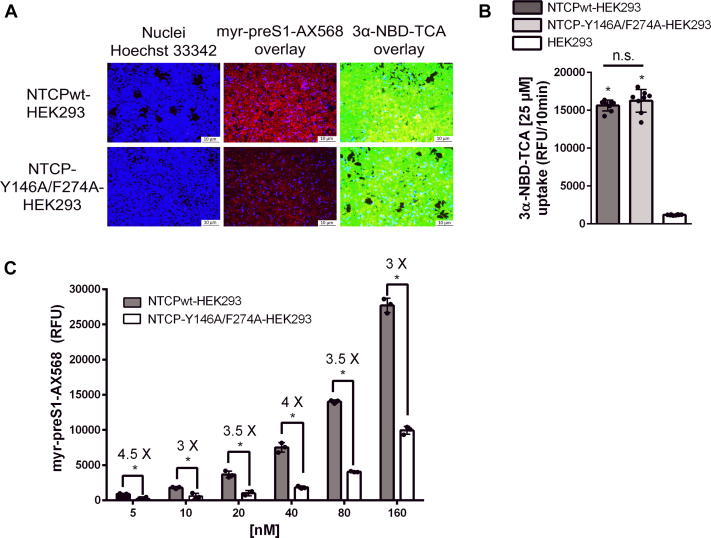
Bile acid transport function and myr-preS1 peptide binding of the NTCP-Y146A/F274A mutant. (A) Bile acid transport experiments with 3*α*-NBD-TCA at 25 *μ*M over 10 minutes at 37 °C and binding of the fluorescently labeled myr-preS1-AX568 peptide at 10 nM for 10 minutes at 37 °C were analyzed in NTCPwt-HEK293 and NTCP-Y146A/F274A-HEK293 cells. Nuclei were stained by Hoechst 33342 (blue). (B) Bile acid transport at 25 *μ*M 3*α*-NBD-TCA over 10 minutes at 37 °C in NTCPwt-HEK293 and Y146A/F274A-NTCP-HEK293 cells. Noncarrier expressing HEK293 cells were used as control. Transport experiments were stopped by removing the transport medium and washing each cell monolayer with PBS. NBD-fluorescence was quantified by GloMax Fluorescence Reader. Values represent means ± SD of quadruplicate determinations of 2 independent experiments. ∗Significantly higher 3*α*-NBD-TCA transport compared with control according to two-tailed unpaired *t* test with *P* < .01. (C) Concentration-dependent binding of the fluorescent myr-preS1-AX568 peptide to NTCPwt-HEK293 and NTCP-Y146A/F274A-HEK293 cells at concentrations ranging from 5 to 160 nM at 37 °C. Values represent means ± SD of quadruplicate determinations. ∗Significantly lower binding to the NTCP-Y146A/F274A mutant according to two-way ANOVA with *P* < .01.

Both cell lines, namely NTCPwt-HEK293 and NTCP-Y146A/F274A-HEK293 cells, were also screened for bile acid transport function with the fluorescent bile acid derivative 3*α*-NBD-TCA^38^ as the probe substrate as well as for myr-preS1 binding competence with the fluorescent myr-preS1-AX568 derivative as surrogate parameter for HBV virus binding to NTCP. As shown in Fig. 4A, both cell lines showed comparable accumulation of 3*α*-NBD-TCA but myr-preS1-AX568 revealed a much lower binding to the NTCP-Y146A/F274A mutant compared with NTCPwt. This effect was closer analyzed by transport experiments with 3*α*-NBD-TCA (Fig. 4B) and binding experiments with myr-preS1-AX568 (Fig. 4C). The NTCP-Y146A/F274A mutant displayed transport rates for 3*α*-NBD-TCA (25 *μ*M) comparable to NTCPwt, indicating that the Y146A/F274A substitution does not compromise the bile acid transport function of NTCP (Fig. 4B). In contrast, binding studies using the AX-568-labeled fluorescent myr-preS1 exhibited significantly reduced binding to the Y146A/F274A NTCP mutant over concentrations ranging from 5 to 160 nM. This clearly underscores the functional importance of Y146 and F274 for myr-preS1 binding (Fig. 4C).

### 3.4 Experimental validation of the lower binding capability of the W41G mutant myr-preS1 peptide

In the next step, the role of the myr-preS1 amino acid W41 for the interaction with NTCP was analyzed. For these experiments a wild-type myr-preS1 peptide of genotype D was used as well as a W41G mutant of this myr-preS1 peptide. Of note, the myr-preS1 peptide of genotype D is highly conserved with bulevirtide at all amino acid positions that showed dominant interactions with NTCP (see Fig. 2A). However, as all subsequent virology experiments were performed with HBV virus particles of genotype D, we decided to use the respective myr-preS1 peptide at this state of the study instead of bulevirtide.

**Fig. 5:**
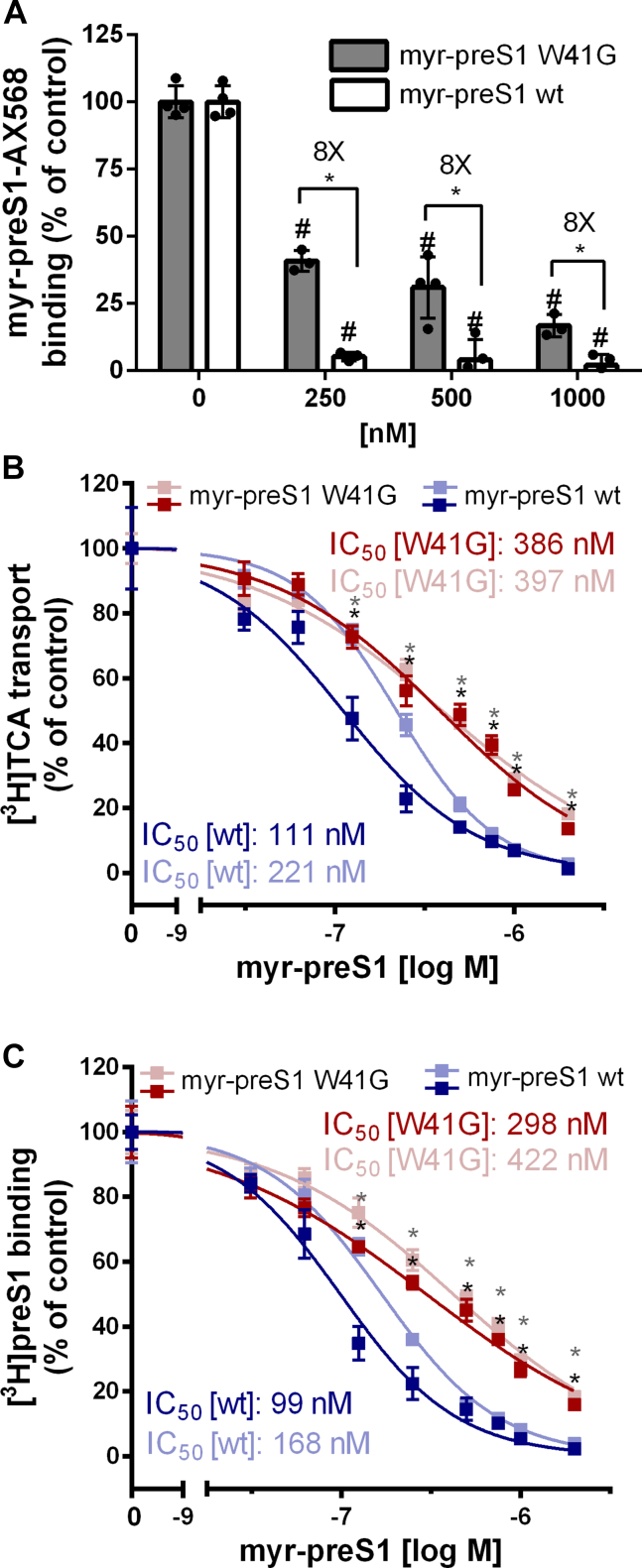
Inhibition of the transport and receptor function of NTCP with the wild-type and W41G mutant myr-preS1 peptide. (A) NTCP-HEK293 cells were preincubated for 20 minutes at 37 °C with the wild-type myr-preS1 peptide (genotype D) or the W41G mutant myr-preS1 peptide at 250, 500, or 1000 nM concentrations. Then, the fluorescently labeled myr-preS1-AX568 was added (10 nM, incubated for 20 minutes at 37 °C) to analyze receptor binding. Fluorescence was detected with the Spark fluorescence reader (excitation: 570 nm, emission: 615 nm). Data represent means ± SD of quadruplicate determinations. ∗Significant difference between the wild-type myr-preS1 peptide and the W41G mutant, and ^#^significantly different to control (without myr-preS1) according to two-way ANOVA with *P* < .01. (B) Systematic analysis of myr-preS1 peptide binding to NTCP with the wild-type (blue curves) and W41G mutant (red curves) peptides, respectively. The transport function of NTCP was analyzed with 1 *μ*M [^3^H]TCA as the substrate over 10 minutes at 37 °C and the myr-preS1 peptides were used as transport inhibitors. (C) In parallel, 5 nM [^3^H]preS1 was used as binding ligand to the NTCP receptor over 10 minutes at 37 °C and the myr-preS1 WT (blue curves) and W41G mutant (red curves) peptides were used for competitive replacement. All experiments were performed in 96 well plates and NTCP wild-type expression in the HEK293 cells was induced by tetracycline treatment. Cells without tetracycline treatment were used as 0% controls of [^3^H]TCA uptake and [^3^H]preS1 binding, respectively. IC_50_ values were calculated after nonlinear regression analysis. In both diagrams (B and C) IC_50_ curves from 2 independent experiments are shown. Data represent means ± SD of quadruplicate determinations. In B and C, asterisks indicate significantly lower inhibition for the W41G mutant myr-preS1 peptide compared with the wild-type myr-preS1 peptide based on two-way ANOVA with *P* < .01.

As shown in Fig. 5A, the wild-type myr-preS1 peptide was able to completely abolish binding of the fluorescent myr-preS1-AX586 peptide derivative to NTCPwt already at 250 nM concentration. In contrast, the mutant myr-preS1-W41G mutant was much less effective in binding to NTCP and did not achieve to fully prevent binding of the myr-preS1-AX568 peptide at 10 nM to NTCP, even not at very high concentrations of 1000 nM. This indicates that W41G mutation of myr-preS1 dramatically reduces the binding affinity to NTCP. This effect was validated by more sophisticated experiments that quantitatively measured transport of radiolabeled TCA ([^3^H]TCA) via NTCP (Fig. 5B) and binding of a radiolabeled myr-preS1 peptide of genotype D ([^3^H]preS1) to NTCP (Fig. 5C). In both experimental setups that were applied in 2 independent experiments, increasing concentration of either the wild-type myr-preS1 peptide or its W41G mutant were used as inhibitors of NTCP at increasing concentrations. As shown in Fig. 5B, the wild-type myr-preS1 peptide revealed much lower IC_50_ values of 111 and 221 nM compared with the W41G myr-preS1 mutant with IC_50_ values of 386 and 397 nM to inhibit [^3^H]TCA transport. In the same line, the wild-type myr-preS1 peptide blocked the binding of the [^3^H]preS1 peptide to NTCP with higher affinity (IC_50_ of 99 and 168 nM) compared with the mutant myr-preS1 W41G peptide (IC_50_ of 298 and 422 nM) (see Fig. 5C). Overall, these experiments demonstrate that by W41G mutation the binding affinity of the myr-preS1 peptide to NTCP is significantly reduced.

### 3.5 Experimental validation of the significant role of the NTCP Y146/F274–myr-preS1 W41 interaction site for in vitro HBV infection of HepG2 hepatoma cells

**Fig. 6:**
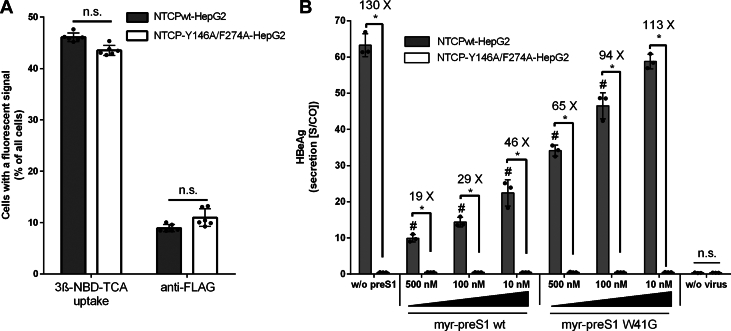
In vitro HBV infection of NTCP-transfected HepG2 cells. HepG2 cells were transiently transfected with either NTCPwt or the Y146A/F274A-NTCP mutant. This transfection was performed in 3 independent experiments, which varied in their absolute transfection rates. Therefore, representative data from 1 out of 3 independent experiments are shown, rather than combined data from all experiments. (A) To assess functional NTCP expression after transfection, transport studies were performed with the fluorescent bile acid 3*β*-NBD-TCA and anti-FLAG immunofluorescence was used to directly detect the expressed wild-type and mutant NTCP proteins. For both assays, the number of fluorescent cells was quantified by automated image analysis on 6 different fluorescent images. Data represent means ± SD and statistical significance was analyzed by Student’s *t* test with *P* < .05. (B) In vitro HBV infection of NTCPwt- and Y146A/F274A-NTCP-transfected HepG2 cells and inhibition with the wild-type (genotype D) myr-preS1 peptide or its W41G mutant at increasing concentrations. Before HBV infection, NTCP-transfected HepG2 cells were preincubated with 10, 100, or 500 nM of the respective myr-preS1 peptides for 1 hour. Subsequently, cells were infected with cell-culture-produced HBV (subgenotype D3) derived from a stably transfected inducible HepG2 cell line as reported.^40^ Cells were incubated with the virus inoculum and the respective myr-preS1 peptide for 24 hours. After infection, the cell culture medium was renewed every 2 to 3 days. Using the supernatants collected on day 10 post infection, HBeAg secreted by infected cells was quantified. Data represent means ± SD of triplicate determinations. ∗Significant difference between the NTCPwt- and the NTCP-Y146A/F274A-expressing HepG2 cells and ^#^significant inhibition in the NTCPwt-HepG2 cells compared with control (without preS1) with *P* < .05 according to two-way ANOVA.

Finally, it was analyzed whether the Y146A/F274A mutant of NTCP and the W41G mutant of myr-preS1 affected the in vitro infection of NTCP-transfected HepG2 cells with HBV virus particles. HepG2 cells were transiently transfected with either NTCPwt or the Y146A/F274A double mutant NTCP construct, respectively. Functional NTCP expression was then analyzed by NBD-TCA transport studies and anti-FLAG immunofluorescence, comparable to the experiments in HEK293 cells (see Fig. 3A and 4A). As shown in Fig. 6A, about 45% of all cells accumulated NBD-TCA without any difference between the NTCPwt and the NTCP-Y146A/F274A mutant constructs. In about 10% of all cells, NTCP expression was detected by anti-FLAG immunofluorescence, again with no difference between the NTCPwt and the NTCP-Y146A/F274A mutant constructs. The differences between the 2 counts (NBD-TCA transport and anti-FLAG immunofluorescence) could be explained by active NBD-TCA transport even by lower amounts of NTCP expression that were not yet detected by immunofluorescence. Then, the NTCP-transfected HepG2 cells were infected with HBV virus particles of subgenotype D3, in the absence (without preS1) or presence of increasing concentrations of the myr-preS1 wild-type or W41G mutant peptides, each at 10, 100, or 500 nM concentrations, respectively. As shown in Fig. 6B, HepG2 cells expressing the NTCP-Y146A/F274A double mutant were completely resistant to in vitro HBV infection at all experimental conditions. This clearly underscores the significant role of the NTCP amino acids Y146 and F274 for HBV virus binding. As expected, and previously reported,^25^ coincubation of the HBV virus particles with the wild-type myr-preS1 peptide significantly suppressed in vitro HBV infection in a concentration-dependent manner at all tested concentrations of 10, 100, and 500 nM. Even at the lowest myr-preS1 concentration of 10 nM, the amount of secreted HbeAg was significantly reduced. In contrast, 10 nM of the W41G mutant myr-preS1 peptide were not able to significantly reduced HbeAg secretion and the highest concentration of 500 nM myr-preS1 W41G peptide reduced the amount of secreted HbeAg only by half, clearly supporting lower binding affinity of the myr-preS1 peptide when amino acids W41 is mutated to glycine.

## 4 Discussion

### 4.1 Y146/F274 as potential target site of NTCP for virus-selective HBV entry inhibitors

In the present exploratory study, we aimed to identify domains at the surface of NTCP that might be appropriate for structure-based drug design to develop virus-selective HBV entry inhibitors. Based on binding energy calculations and MD simulations, both using the recent cryo-EM structure of the NTCP-bulevirtide complex,^35^ we identified a binding triangle between Y146 (EL2) and F274 (TM8b) of NTCP with W41 of the myr-preS1 string structure. Even if the short run time of the MD simulations of 100 ps is a limitation of this part of the study, this was only used as an additional tool to select the appropriate amino acids for the experimental mutation studies. The strong interaction site between Y146 and F274 of NTCP with W41 of the myr-preS1 peptide is based on *π–π* and HIs between the aromatic rings of the respective amino acid side chains. Such *π–π* interactions, also called aromatic interactions, form strong noncovalent intermolecular forces that are involved in the stabilization of DNA and protein structures, as well as in drug binding.^41^ We clearly demonstrate by experimental mutation studies that the Y146/F274 binding site at NTCP is essential for virus binding and in vitro HBV infection, whereas W41 contributes to the high affinity binding of the myr-preS1 peptide to NTCP. Of note, W41 is highly conserved between all human HBV genotypes A–I and is also present in the drug bulevirtide. In addition, Y146 and F274 are highly conserved between all NTCP sequence of human, apes, OWM, new world monkeys, mouse, rat, dog, pig, horse, cattle, and tree shrew (see Fig. 1C). Based on this, the Y146/F274 domain of NTCP can be used as the center for virtual structure-based drug design that might help in the future to identify virus-selective HBV/HDV entry inhibitors acting via NTCP inhibition. A potential limitation of this finding is that Y146 and F274 are located on the surface of NTCP, where a well defined binding pocket is absent for direct structure-based drug design. As a result, this site may not be directly druggable. Nevertheless, an alternative approach could be to target an adjacent cavity with high-affinity ligands and to design a side chain that sterically blocks the binding of myr-preS1 to the Y146/F274 site on NTCP, thereby inhibiting the virus–receptor interaction. Importantly, based on the data presented here and the available cryo-EM structures of the bulevirtide–NTCP complex, viable alternatives for the structure-based design of a virus-selective HBV entry inhibitor appear extremely limited, if they exist at all. At the current state of knowledge, given the challenges associated with targeting the NTCP Y146/F274–myr-preS1 W41 interface, it cannot be excluded that a structure-based approach to develop a virus-selective HBV entry inhibitor may ultimately prove unsuccessful.

### 4.2 Other potential target sites of NTCP for nonvirus-selective HBV entry inhibitors

It is important to emphasize that precise localization of an NTCP inhibitor binding site is critical for distinguishing between inhibitors that also block the binding and transport of physiological bile acid substrates (nonselective HBV entry inhibitors) and those that bind at more distant sites, thereby preserving bile acid transport (virus-selective HBV entry inhibitors).

Based on recent cryo-EM structural information NTCP has 2 separated domains, named core domain (comprising TM 2–4 and TM 7–9) and panel domain (consisting of TM 1, 5, and 6).^30^, ^31^, ^32^, ^33^, ^34^, ^35^ Together, these domains form a continuous tunnel structure that serves as the bile acid transport pathway.^32^ This tunnel-like structure provides multiple interactions sites with the myr-preS1 plug. The myr-preS1 plug includes the _9_NPLGFFP_15_ motif that has been identified to be essential for virus binding and infectivity and is highly conserved among the HBV genotypes A–J.^3^^,^^4^ According to the structural data of the myr-preS1/NTCP complex, this motif deeply penetrates the NTCP transport pore and directly interferes with the bile acid substrate binding sites of NTCP^32^ (see Fig. 1). Even if the amino acids flanking the _9_NPLGFFP_15_ motif do not directly interfere with the bile acid substrate binding site at NTCP, they completely clog the entrance into the tunnel structure, so that bile acids cannot reach their substrate binding site. As a result, any inhibitor designed to block myr-preS1 binding within this tunnel would inevitably also impede bile acid transport, leading to nonselective NTCP inhibition. Hence, despite the strong interaction observed between the myr-preS1 _9_NPLGFFP_15_ motif and the tunnel site of NTCP (see Fig. 2), this region was not pursued further as a viable target site for the development of a virus-selective NTCP inhibitor in the present study.

This is exemplified by the genetic c.800G>A variant (rs2296651) that is frequent in East Asian populations and that leads to S267F mutation of NTCP inside the tunnel structure. This variant shows a nearly complete loss of its bile acid transport function^42^^,^^43^ and has been associated with decreased risk of HBV infection.^44^, ^45^, ^46^ On the other hand, the _157_KGIVISLVL_165_ domain of NTCP that also interacts with the myr-preS1 peptide^3^^,^^47^^,^^48^ could be an additional potential target site for virus-selective NTCP inhibition, as we have previously suggested.^39^ More detailed analysis of this region revealed that G158 (TM 5) is a critical position for myr-preS1 binding and HBV infection. It is well established that OWM, bearing an arginine at this position are nonsusceptible to HBV infection. However, R158G mutation of OWM Ntcp was sufficient to support myr-preS1 binding an in vitro HBV and HDV infection.^39^ Interestingly, G158 has been identified as a rheostat position with a wide range of transport activities depending on the amino acid substitution,^43^ but as a toggle switch position for the virus receptor function of NTCP, with only glycine as the smallest amino acid supporting myr-preS1 binding.^49^ Of note, the 158R NTCP variant found in OWM is resistant to myr-preS1 binding while preserving fully active bile acid transport.^39^ This suggests that G158 could, in theory, represent an additional potential target site for structure-based drug design. However, we observed no significant direct molecular interactions between G158 and NTCP. Instead, the G158R substitution appears to sterically block myr-preS1 binding.

### 4.3 Lessons learnt from the recent NTCP cryo-EM structures as basis for drug development

Our study is based on the recent breakthrough in structural determination of the myr-preS1/NTCP receptor complex. The first structure was generated from an NTCP lacking the 29 C-terminal amino acids and myr-preS1 of HBV genotype B.^34^ This study used 2 different Fab fragments from mouse monoclonal antibodies to stabilize the myr-preS1/NTCP complex and resolved 2 basically identical structures of 2.9 Å (NTCP-myr-preS1-YN9048Fab, PDB 8HRX) and 3.1 Å (NTCP-myr-preS1-YN9016Fab, PDB 8HRY) resolution.^34^ This structure resembled the apo-NTCP structure in the outward-facing conformation^30^ (PDB 7VAD) with an overall RMSD value of 1.33 Å.

The second cryo-EM structure was resolved with 3.4 Å from a complex of the drug bulevirtide (representing the amino acids 2–48 of the myr-preS1 peptide of HBV genotype C) and the full-length human NTCP^35^ (PDB 8RQF) that was stabilized with a specific antigen-binding antibody fragment (Fab3) obtained from a synthetic library using phage display. This structure of the bulevirtide/NTCP complex only showed an overall RMSD of 0.767 Å with the substrate-bound open pore structure of NTCP^32^ (PDB 7ZYI). Thereby, the panel TM1, 5 and 6 are slightly stretched due to bulevirtide binding. Within this structure, residues 11 to 13 of bulevirtide directly overlap with the outer bile acid binding site so that simultaneous binding of myr-preS1 and bile acids is excluded.

The structures of both studies^34^^,^^35^ have quite similar conformations with an overall RMSD of 0.496 Å. Even if the myr-preS1 peptides used for cryo-EM differ somewhat in their amino acid sequence (genotype B^34^ and genotype C-based bulevirtide,^35^ respectively), the myr-preS1 structure revealed basically the same fold. Based on this, we assume that also the HBV myr-preS1 peptide of genotype D as used in the present study, has the same binding structure. However, the domain assignment of the myr-preS1 peptide was somewhat different between both studies. In the study by Asami et al,^34^ a lasso-like structure (representing the N-terminal 2–34 amino acids of myr-preS1) were classified to interacts with the tunnel structure of NTCP, whereas the C-terminal 35 to 48 amino acids interact with the surface of NTCP. In the study by Liu et al,^35^ based on structural observations the bulevirtide peptide was separated into a plug structure (amino acids 2–20) that deeply penetrates the NTCP translocation tunnel and a string structure (amino acids 21–48) that covers the surface of NTCP. As the initial MD simulations of the present study were based on the bulevirtide/NTCP complex, we used the partition according to the study by Liu et al,^35^ however, regarding the identification of an interaction domain at the surface of NTCP without any interference with the transport tunnel, the focus was on the C-terminal 35 to 48 amino acids of myr-preS1 in the second part of the present study.

In both above mentioned cryo-EM structures,^34^^,^^35^ plenty of molecular interactions have been identified. However, interaction energies have not been determined so far. Based on this structural information, the present study aimed to identify molecular interaction hot spots with high binding energies based on MD simulations. The numerous contact sites between NTCP and myr-preS1/bulevirtide that have been proposed in these structural studies also included the high-energy molecular interaction sites A–C identified in the present study by means of MD (see Fig. 1E).^35^

Interaction site A is formed between F13 and F14 of myr-preS1 and the amino acids Q264, F283, F284, and L287 (TM8b, EL4, and TM9) as well as F18 (TM1) of NTCP; interaction sites B between F23 of myr-preS1 and V29 and V32 of NTCP; and interaction site C between K38, D39, H40, and W41 of myr-preS1 and A273, F274, and P275 (TM8b, EL4, and TM9) as well as Y146 (of EL2) of NTCP. For some of these amino acids, mutation studies confirmed their role for the myr-preS1 interaction. As an example, Q264A mutation of NTCP retained bile acid transport functions but showed significantly reduced myr-preS1 binding, indicating that Q264 is important for preS1 binding and HBV infection.^34^ However, as Q264 mutation to the bulky tryptophan residue (Q264W) completely abrogated bile acid transport, Q264 is supposed to be close to the bile acid binding and translocation pathway, which supports our conclusion that interaction site A is not appropriate for structure-based drug design for virus-selective inhibitors as it is too close to the bile acid translocation tunnel. Amino acid V272 that is flanking interaction site C was also mutated in the previous study by Asami et al.^34^ V272W mutation showed intact bile acids transport but significantly reduced myr-preS1 binding and HBV infection, suggesting that V272 is involved in myr-preS1 binding and HBV infection, but not in bile acid translocation. In addition, F274A single mutation caused a reduction of HBV preS1 susceptibility to host cells. Amino acid F274 has also been suggested to be involved in NTCP oligomerization.^50^ Both findings support the suitability of interaction site C as target site for virus-selective NTCP inhibitors. Of note, the effect found for V272W mutation is such as the effect of Y146A/F274A mutation in the present study, both showing intact bile acid transport but reduced binding affinity for the myr-preS1 peptide.

Even if the NTCP domain covering residues _84_RLKN_87_ in human NTCP (TM2-TM3) has been identified as relevant for myr-preS1 binding before,^47^^,^^51^ this domain was not considered in the present study due to low predicted binding energies. However, this domain also covers the surface of NTCP and not the tunnel structure. Within the published structures N87 forms hydrogen binds with E43 and K46 of the myr-preS1 string structure, while for the NTCP amino acid residues 84 to 86 no interactions with the myr-preS1 peptide could be detected. As mouse Ntcp with the corresponding residues _84_HLTS_87_ has S87 instead of N87 in this position, this interaction was not considered relevant for the present study (see Fig. 1B). Nevertheless, the 84 to 87 humanized construct of mouse Ntcp did not only bind myr-preS1 but also supported infection.^47^^,^^51^ This data indicates that the _84_RLKN_87_ domain of NTCP might be essential to trigger endocytosis of the virus/receptor complex. In addition, based on a recent cryo-EM structure of macaque Ntcp^49^ it was shown that K86 of human NTCP better than N86 of the macaque Ntcp contributes to the myr-preS1 binding to NTCP. However, we did not find significant molecular interaction at this position of the myr-preS1 peptide in the MD simulation of the present study.

## 5 Conclusion

The myr-preS1-W41/NTCP-Y146/F274 interaction site that is characterized by high binding energies is essential for HBV virus entry into hepatocytes. As this domain has some distance to the bile acid binding and translocation site of NTCP, the Y146/F274 domain of NTCP is an attractive receptor domain for structure-based development of virus-selective HBV entry inhibitors that do not disturb the physiological bile acid transport function of NTCP.

## Conflicts of interest

The authors declare no conflicts of interest.
